# De novo reconstruction of satellite repeat units from sequence data

**Published:** 2023-04-19

**Authors:** Yujie Zhang, Justin Chu, Haoyu Cheng, Heng Li

**Affiliations:** 1 Harvard School of Public Health, 677 Huntington Avenue, Boston, MA 02115, USA; 2 Department of Data Science, Dana-Farber Cancer Institute, 450 Brookline Ave, Boston, MA 02215, USA; 3 Department of Biomedical Informatics, Harvard Medical School, 10 Shattuck St, Boston, MA 02115, USA

## Abstract

Satellite DNA are long tandemly repeating sequences in a genome and may be organized as high-order repeats (HORs). They are enriched in centromeres and are challenging to assemble. Existing algorithms for identifying satellite repeats either require the complete assembly of satellites or only work for simple repeat structures without HORs. Here we describe Satellite Repeat Finder (SRF), a new algorithm for reconstructing satellite repeat units and HORs from accurate reads or assemblies without prior knowledge on repeat structures. Applying SRF to real sequence data, we showed that SRF could reconstruct known satellites in human and well-studied model organisms. We also found satellite repeats are pervasive in various other species, accounting for up to 12% of their genome contents but are often underrepresented in assemblies. With the rapid progress on genome sequencing, SRF will help the annotation of new genomes and the study of satellite DNA evolution even if such repeats are not fully assembled.

## 1 INTRODUCTION

Satellite DNA (SatDNA) are long tandemly repeating sequences that look like “BBBBBB···”, where each symbol “B” represents a repeat unit, also known as a *monomer*. A monomer “B” could range from a few basepairs (bp) to thousands of bp in length and an entire SatDNA could span megabases in large genomes. Several percent of the human genome, or a couple of hundred megabases in total, is composed of SatDNA (Altemose et al., 2022). Monomers in a SatDNA array are similar in sequence but often not identical due to random mutations.

In some species, SatDNA may be organized as high-order repeats (HORs; Miga 2019). For example, the centromere of human chromosome 2 has a pattern like “ABCDABCDABCD···”. Letters “A”–“D” correspond to four diverged alpha repeat monomers of ~171bp each, respectively, and the “ABCD” unit is repeated many times in the centromere with all copies being similar to each other. Researchers who study centromere repeats usually say “ABCD” is a 4-mer HOR unit. Because we will often mention short nucleotide sequences in this article, we will call “ABCD” in this example as 4-monomer HOR unit to avoid confusion.

SatDNA is often not assembled in long contigs due to its repetitiveness. We would have to reconstruct SatDNA from raw sequence reads in this case (Lower et al., 2018). Wei et al (Wei et al., 2014) developed k-Seek to study SatDNA consisting of 2–10bp repeat units. Melters et al (Melters et al., 2013) applied Tandem Repeat Finder (TRF; Benson 1999) to Sanger reads and fragmented short-read contigs to find the most common monomer in each species. TAREAN (Novák et al., 2017) does all-vs-all comparison between short reads, clusters the reads and then identifies circular structures from the cluster graphs. These methods can reconstruct unknown monomers but they are unable to reveal HOR structures. On the contrary, Alpha-CENTAURI (Sevim et al., 2016) reconstructs HORs from long reads but it requires known monomer sequences.

With improved sequencing technologies, it is now possible to assemble through human centromeres (Nurk et al., 2022). More recent methods, including NTRprism (Altemose et al., 2022), HORmon (Kunyavskaya et al., 2022) and HiCAT (Gao et al., 2022), can identify detailed chromosome-specific HOR patterns from complete SatDNA sequences in human. These methods demand high-quality assembly and rich prior knowledge on SatDNA in the studied species. However, the finished human genome, CHM13, was derived from a near homozygous molar cell line that is easier to assemble. For a normal diploid human individual, we could only assemble through a fraction of SatDNA even with the best possible data and algorithm (Rautiainen et al., 2023). The complete assembly of other species is even rarer. This has limited the application of such assembly-based SatDNA reconstruction algorithms.

In this article, we will describe a new algorithm, Satellite Repeat Finder (SRF), for assembling SatDNA repeat units. SRF overcomes the limitation of previous methods. It is applicable to both accurate reads and high-quality assembly and is able to automatically reconstruct HORs with no prior knowledge on monomer sequences.

## 2 RESULTS

### 2.1 The SRF algorithm

In a SatDNA array “BBBBBB···”, suppose every monomer “B” is identical to each other. Under a long enough , the *k*-mer de Bruijn graph of the SatDNA array will be a single cycle. When there are basepair differences between monomers, the de Bruijn graph will not be a simple cycle. If there are many different monomers, the de Bruijn graph can become very complex and cannot be resolved with classical graph cleaning algorithms (Zerbino and Birney, 2008).

Our intuition is that if there are many copies of the monomer, we may still be able to find a cycle composed of highly abundant *k*-mers in the de Bruijn graph. We can start with the most abundant *k*-mer and at each bifurcation in the graph, we greedily choose the *k*-mer of the highest occurrence. We repeat this process until we go back the starting *k*-mer, which will reconstruct a repeat unit, or come to a deadend, which will be discarded. Algorithm 1 provides more details. Here, indicates *k*-mer and are adjacent in the de Bruijn graph. For simplicity, this algorithm traverses a unidirected de Bruijn graph. In SRF, we implemented a bidirected de Bruijn graph such that we will not find a repeat unit on both strands.

SRF works with Illumina short reads, PacBio HiFi long reads and high-quality assembly contigs and can identify HORs (Table 1). When assembling satellite repeats from PacBio HiFi reads in this article, we counted 151-mers with KMC (Kokot et al., 2017) and collected 151-mers occurring ≥10 times over the average read coverage. K-mer counting may take a few tens of minutes for a high-coverage human dataset and is the performance bottleneck. SRF only takes seconds to reconstruct all repeat units after k-mer counting.

### 2.2 Estimating satellite abundance

The SRF algorithm does not provide a good estimate of repeat abundance. We mapped all input sequences against reconstructed repeat units to measure the total length of each repeat unit. For human CHM13 data, we observed many diverged hits between HORs and scattered monomers in pericentromeric regions. We thus filtered hits of identity below 90% to get more accurate HOR length estimates. The effect of the identity is determined by the repeat structure in a species. For example, switching off the filter would increase the total abundance estimate by 40% for human but only by 4% for *A.thaliana*. We still applied this filter to all datasets even though this may lead to underestimates for some species.

SRF may reconstruct repeat units similar in sequence. The similar repeat units may be mapped the same genomic locus. To remove redundancy, we only select the hit of the highest identity among hits overlapping on an input sequence. With this procedure, we map each base on an input sequence to at most one repeat unit.

Occasionally a small number of long terminal repeats (LTRs) may occur tandemly in a few region. SRF may identify such LTRs even though they do not form long tandem arrays. When estimating abundance, we additionally filter out repeat with <2 tandem copies in the middle of a sequence or with <1.5 tandem copies when the repeat-to-read alignment reaches the end of a read. This filter is reliable when we apply SRF to assemblies but may miss long repeat units when applied to reads. We again opted for conservative estimates.

### 2.3 A brief introduction to human satellites

In the human genome, the most abundant satellite family is alpha satellites with most of them present in long alpha HORs (). The consensus of the minimal alpha repeat unit is 171bp in length. The active centromeric regions that centromeric proteins bind to are primarily composed of (Altemose et al., 2022). Conversely, though, not all are present in the active regions. These inactive tend to be shorter than active ones. Alpha repeat monomers are also present in pericentrometic regions without clear HOR structures. In addition to alpha repeat, the human genome is also enriched with three types of human satellites (HSat1–3), contributing to a few percent of human genome (Altemose, 2022). Almost all these satellites are located around centromeres or on the long arm of the Y chromosome.


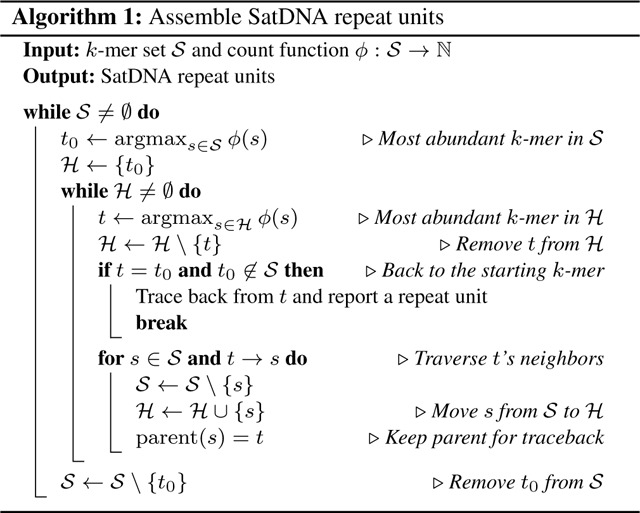


In the human reference genome GRCh38 (Schneider et al., 2017), *α*HORs were computationally generated from a Hidden Markov Model (Miga et al., 2014); HSats are underrepresented. At present, only the T2T-CHM13 assembly (Nurk et al., 2022) provides a complete representation of all satellite arrays.

### 2.4 Annotating satellite repeats in human T2T-CHM13

We first ran SRF on each T2T-CHM13 chromosome separately and compared the results to existing annotations by HORmon (Kunyavskaya et al., 2022) and HiCAT (Gao et al., 2022). HORmon reports the same *α*HOR lengths as Altemose et al. (2022). In Table 2, column “SRF (*k*=171) chromosome” shows the lengths of HORs identified by running SRF on individual chromosomes. SRF reported the same *α*HOR lengths as HORmon except for chromosome 5, 8, 9, 13 and 18. The chromosome 8 of T2T-CHM13 has been well studied by Logsdon et al. (2021). Although the 11-monomer is the most abundant, it is interleaved with 4-, 7- and 8-monomers that are derived from the 11-monomer. The 7-monomer is the second most abundant array and forms the longest *α*HOR array in the middle of the centromere. The greedy SRF algorithm chooses the 7-monomer over the 11-monomer possibly because the 7-monomer has a more conservative consensus. The SRF-HORmon differences in other chromosomes may have a similar cause.

Unlike HORmon and HiCAT which require users to provide the monomer sequence and prepare centromeric sequences, SRF was directly applied to whole chromosome sequences with no prior knowledge. In addition to active , SRF identified shorter *α*HOR arrays outside the active regions. It also found many long non-alpha satellite arrays including a repeat unit of 1,814 bp on chr15, of 6,112 bp on chr16, of 3,569 bp on chrY and of 2,420 bp on chrY as well. These span over one megabase and have been reported previously (Altemose, 2022).

SRF further found a satellite array on the long arm of chromosome 1 between coordinate 227,746,662 and 228,024,151. The repeat unit is 2,240 bp in length, composed of an AluY repeat, a 5S-RNA and di-nucleotide repeats. This is the only non-centromeric array in T2T-CHM13 longer than 100kb.

The SRF inference on the whole T2T-CHM13 genome (column “SRF/171 assembly” in Table 2) is close to the inference on individual chromsomes. SRF missed the *α*HOR array on chr14 and chr21 because chr22 and chr13, respectively, have very similar arrays which are merged during the whole-genome inference.

SRF works on sequence reads which HORmon, HiCAT and NTRprism are not applicable to. On PacBio High-Fidelity (HiFi) reads, SRF reconstructed similar to the whole-genome reconstruction (column “SRF/171 HiFi reads”). It can also identify the majority of from Illumina short reads (column “SRF/101 Illumina” in Table 2), though the use of shorter 101-mer reduces the sensitivity to some arrays.

From Table 2 we can see that some *α*HOR arrays, such as those on chr3 and chr11, can be consistently reconstructed by various tools on different types of input data. However, some other arrays, such as those on chr8 and chr19, are intrinsically harder to reconstruct. These are probably because monomers in a HOR may be connected in different ways, as is shown by Kunyavskaya et al. (2022).

### 2.5 Satellite repeats in multiple human assemblies

We applied SRF independently to each phased human assembly produced by the Human Pangenome Reference Consortium (HPRC). We identified with dna-brnn (Li, 2019), aligned them to the T2T-CHM13 genome and HORmon consensus and manually assigned the to chromosomes based on the similarity to existing annotations. The last column of Table 2 shows the *α*HOR lengths and their frequencies. We consider two are different if they have different lengths and the shorter *α*HOR cannot be aligned into the longer one at <2% sequence divergence.

There are 47 diploid samples and 94 haploid assemblies. We could find assigned to individual chromosomes in most cases. We sometimes see of different lengths assigned to the same chromosome but their sequence divergences are small. This again could be caused by the different ways HOR monomers are connected in individual samples (Logsdon et al., 2021; Kunyavskaya et al., 2022).

We also applied SRF to the pool of all haploid assemblies. This procedure may miss infrequent satellite arrays but it helps to simplify the study of shared arrays. In addition to active , we identified supposedly inactive that are at >10% divergence from existing annotated . Notably, there is a 20-monomer *α*HOR that is mapped to the chromosome 15 of T2T-CHM13 and spans several hundred kilobases in most samples. There are other examples like this. SRF also found long HSat arrays and non-HOR satellites, including those found in CHM13, which are easy to identify as they do not have internal structures.

### 2.6 Satellite repeats in *Arabidopsis thaliana*

We obtained three HiFi datasets (Table 3) and downsampled them to about 40-fold coverage each. Col-0N and Ey15–2R were sequenced from a pool of multiple samples and Col-0R from a single sample.

The *A. thaliana* centromeres are composed of CEN180 satellites which are known to have high-order organizations (Naish et al., 2021). When we pooled the two Col-0 datasets and applied SRF, we reconstructed one 1-monomer, two 2-monomers, one 3-monomer and one 6-monomer HORs. However, this result is unstable. If we ran SRF on each Col-0 dataset separately or on the full coverage, we would get different HORs. We thus ignored the HOR structures and focused on the abundance of CEN180 only.

SRF estimated that 5.2% of read bases in Col-0N and 7.5% in Col-0R are composed of CEN180 satellites. Col-0R has 44% more CEN180 satellites than Col-0N. To check whether this large difference is caused by an artifact, we inferred the relative CEN180 abundance with a different approach as follows. We collected 179-mers matching CEN180 and occurring ≥400 times in the pooled Col-0 dataset. We counted the total numbers of these 179-mers in Col-0N and Col-0R separately and normalized the counts by read bases. We found Col-0R has 41% more CEN180 179-mers than Col-0N, broadly in line with the earlier analysis. Centromere contents may differ greatly even between two samples from the same strain.

**Fig. 1.**
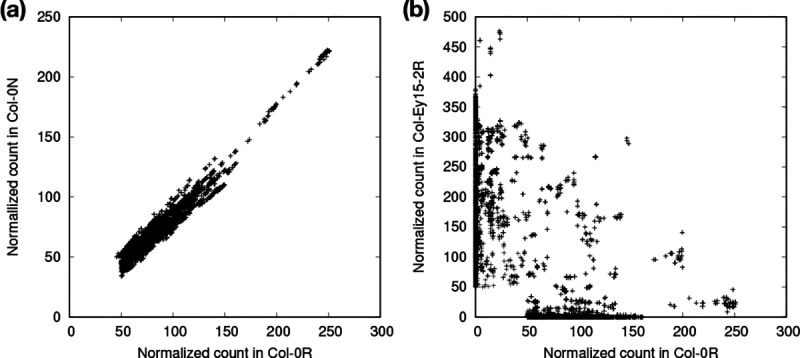
Normalized counts of 179-mers in three *A. thaliana* read datasets. Raw 179-mer counts in reads are normalized by coverage. A 179-mer is selected in the plot if it matches the CEN180 satellite and if its normalized count is at least 50 in one of the datasets. (**a**) Counts between two different samples from the same strain. (**b**) Counts between two different strains.

SRF estimated that 11.5% of Ey15–2R is composed of CEN180 satellites, higher than both Col-0R and Col-0N. Furthermore, while Col-0R and Col-0N share similar high-occurrence 179-mers that match CEN180 (Fig. 1a), Col-0 and Ey15–2 share few common 179-mers (Fig. 1b). The centromere sequences between strains are distinct both in content and in length.

In addition to the CEN180 satellite, SRF also reconstructed a 10,067 bp rDNA unit from the two Col-0 datasets. It has 3.1% abundance in Col-0R and 1.7% in Col-0N. If we assume the *A. thaliana* genome is 132Mb in length according to the Col-0N assembly, Col-0R has ~400 copies of this rDNA unit while Col-0N has ~220 copies. The Col-0N assembly (Naish et al., 2021) only has seven copies, located towards the telomeric ends of chr2 or chr4 short arms. SRF did not reconstruct an rDNA unit from Ey15–2R. We mapped the Col-0 rDNA unit to Ey15–2R reads and estimated that Ey15–2R has ~200 copies.

### 2.7 Satellite repeats in other model organisms

We applied SRF to the HiFi reads of three model organisms (Hon et al., 2020): the reference C57BL/6J strain of *Mus musculus* (mouse; AC:SRR11606870), the F1 generation of the reference ISO1 strain and the A4 strain of *Drosophila melanogaster* (AC:SRR10238607), and the B73 strain of *Zea mays* (maize; AC:SRR11606869).

In mouse, SRF identified two satellite units. The second most abundant repeat is the 234 bp major satellite around centromeres (Arora et al., 2021; Thakur et al., 2021). The first is 1,199 bp in length, composed of 10 copies of the 120 bp minor satellite unit. This confirms the high-order organization of minor satellites observed by Pertile et al. (2009). The full-length hits of this repeat in the mouse reference genome mostly come from the sex chromosomes and are all below 75% in identity. Nonetheless, this repeat is abundant in reads with the majority of alignments at 95% identity or higher. To further investigate this repeat, we assembled the HiFi reads with hifiasm (Cheng et al., 2021). We can find long tandem arrays of this repeat on multiple contigs, all shorter than 1.1Mb. Hifiasm keeps the repeat content but is unable to assemble this satellite.

In *Drosophila*, the most abundant satellite SRF identified is a 358 bp repeat unit hitting 0.90% of read bases. It belongs to the 1.688 family (Khost et al., 2017). The abundance of the 358 bp repeat is lower in the BDGP6 reference genome, at 0.26% only. SRF assembled the 240 bp Intergenic spacer (IGS; Shatskikh et al. 2020) into two sequences, at 240 bp and 239 bp, respectively. The edit distance between the two IGS sequences is 5. They hit to 0.43% of read bases in total but are depleted in the reference at <0.01% only. SRF also found other known satellite repeats such as (AAGAC)n, (AACAC)n, (AATAG)n, (GGTCCCGTACT)n and (AATAACATAG)n (Shatskikh et al., 2020; Thakur et al., 2021). There are more copies of these repeat units but because they are short, they contribute less to the genome in comparison to the 1.688 and IGS satellites.

SRF reconstructed a 5,045 bp repeat unit at 0.34% abundance in reads and 0.08% in the reference genome. It harbors histone genes and is located in a small region on chromosome 2L. To investigate further, we assembled the HiFi reads using the hifiasm trio-binning mode with ISO1 and A4 short reads from SRR6702604, SRR457665, SRR457666 and SRR457707. When aligning the ISO1 haplotype assembly to the reference genome, we see a clean 242kb insertion entirely composed of the 5,045 bp histone repeat. The insertion has 48 tandem copies at >99% identity between the copies. The BDGP6 reference genome might have misassembled this region.

In maize, SRF reconstructed a 741 bp repeat unit at 0.25% abundance. It matches the SAT1_ZM record in RepBase. This SRF unit includes four copies of a 180 bp knob-associated repeat (Ananiev et al., 1998b). In the NAM-5.0 reference genome or the hifiasm assembly, this repeat tends to be present in short contigs and towards ends of long contigs. It is not assembled well. SRF also identified many potential repeat units at <0.09% abundance in reads. Nonetheless, none of them form long tandem arrays. Meanwhile, under the 151-mer setting, SRF failed to identify the 156 bp CentC repeat (Ananiev et al., 1998a). SRF could find this repeat if we counted 101-mers. Only 0.045% of read bases were mapped to CentC. Low-abundance SatDNA is harder to assemble correctly.

### 2.8 Comparison to TAREAN

TAREAN (Novák et al., 2017) can identify novel satellite repeats from sequence reads. Its developers recommend to use reads at up to 0.5-fold coverage to avoid redundancy between reads sequenced from the same loci. We ran TAREAN on simulated short reads at 0.2-fold from the Dropsophila HiFi dataset described above, without introducing additional sequencing errors. TAREAN found six high-confidence satellite repeats, including the 1.688 family and the histone cluster, (GGTCCCGTACT)n and (AATAACATAG)n. The other two TAREAN repeats also hit to SRF contigs. SRF assembled eight more SatDNA repeat units at >0.05% abundance. Manually inspecting the alignment of SRF contigs to raw HiFi reads, we observed tandem pattern for all of them, suggesting they were real SatDNA.

To evaluate whether TAREAN can reconstruct HORs, we ran TAREAN on 0.2-fold CHM13 reads randomly sampled from SRR2088062. TAREAN took 5 hours and found four high-confidence satellite repeats, including a 2-monomer alpha repeat at 1.0% abundance, a HSat2 repeat at 0.9%, a SAR satellite and a beta satellite. TAREAN did not identify other HORs.

### 2.9 Satellite repeats in other species

**Fig. 2.**
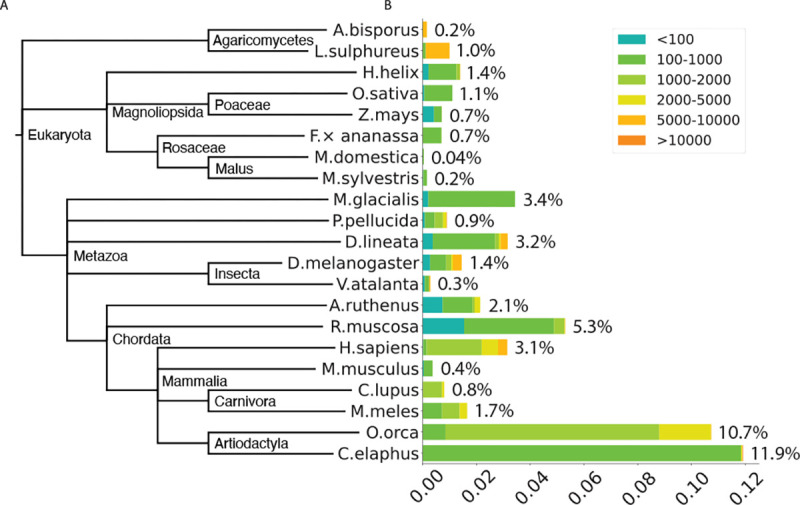
Abundance of satellite DNA in 21 species.

We randomly selected 14 species from the Darwin Tree of Life project and collected two species from Hon et al. (2020) (Table 4). We assembled SatDNA in these and several other species described in earlier sections. SRF may reconstruct mitochondria or chloroplast from sequence reads. We manually removed them based on NCBI BLAST against the nt database. We then estimated the abundance of SatDNA in each of these species (Fig. 2).

Red deer (*C. elaphus*) has the highest abundance at 11.9%. A single 796 bp repeat unit accounts for 10.3% of satellite DNA. Killer whale (*O. orca*) in the same order is also enriched with satellite DNA. Yellow-legged frog (*R. muscosa*) is next to killer whale. SRF reconstructed many distant variants of a 131 bp repeat unit. On the other extreme, apples (*M. domestica* and *M. sylvestris*) barely have satellite repeats partly because they have transposonrich centromeres (Zhang et al., 2019). Plants and fungi are generally depleted of satellites.

It is worth noting that our abundance estimate may be an underestimate due to the additional filters we used. For example, chicken mushroom (*L. sulphureus*) had a repeat unit of 9,659 bp at 0.9% abundance. As we discarded alignments shorter than 1.5 times 9,659 bp, we filtered out many HiFi reads shorter than this threshold even if entire reads were aligned to the repeat. The abundance estimate would be doubled without this filter. Such long repeat units are infrequent in the species we studied.

To investigate what satellites are organized as HOR, we ran TRF (Benson, 1999) on SRF-assembled repeat motif. A repeat motif is considered to have a high-order structure if TRF identifies a tandem repeat repeating at least three times and covering 90% of the motif. With this criterion, 98.5% of human satellites are HORs with a variety of number of monomers. 8.6% of satellites in Eurasian badger (*M. meles*) are HORs of a 138 bp monomer, contributing to 0.14% of the genome. The other species in our survey either do not have multiple HORs composed of similar monomers or only have HORs at <0.1% abundance. Consistent with our observation, Melters et al. (2013) rarely identified HORs consisting of ≥3 monomers. The authors attributed this to the limited Sanger read length. Based on longer reads and a different algorithm, our result suggests that most species do not exhibit rich HOR structures.

## 3 DISCUSSIONS

SRF is a de novo assembler for reconstructing SatDNA repeat units and can identify most known HORs and SatDNA in well-studied species without prior knowledge on monomer sequences or repeat structures. It is the only de novo algorithm for reconstructing HORs from sequence reads as well as high-quality assemblies. SRF only depends on a third-party k-mer counter. It is easy to run and fast to execute.

SRF uses a greedy algorithm to assemble SatDNA repeat units. When two repeat units share long similar sequences, the one of lower abundance and higher diversity may be missed. We plan to improve the current algorithm by reporting multiple overlapping cycles. This may be able to find a more complete collection of HORs in the human genome.

Meanwhile, although SRF can reconstruct known HORs in human, it may report incidental HORs in species, such as mouse and *A. thaliana*, that only have weak high-order patterns. We need to run TRF (Benson, 1999) on SRF contigs to obtain minimal repeat units. SRF may also assemble the same class of repeat into multiple similar but not identical copies. We can align assembled repeat units to identify such redundancy. Manual curation is recommended for a deeper insight into the SatDNA structure of a new species.

Estimating the abundance of SatDNA is challenging. Sometimes ancient SatDNA repeats may be too diverged from the assembled repeat consensus to be aligned confidently. In human, whether to count scattered monomers in pericentromeric regions as long SatDNA arrays would affect the estimate as well. In addition, occasionally SatDNA units can be >5 kb in length. We may not observe clear tandem patterns in ~10 kb HiFi reads, which would lead to underestimate. We do not have an automated algorithm to provide accurate abundance estimate in corner cases.

SatDNA is pervasive in many species. It is however often underrepresented in current reference genomes such as the human GRCh38 genome and the *Drosophila* BDGP6 genome. Even with improved sequencing technologies and assembly algorithms, the assembly of SatDNA is often fragmented. With thousands of species sequenced recently (Challis et al., 2020; Rhie et al., 2021) and more to come in future, SRF may become an important tool to identity and annotate SatDNA in these species. It may also supplement RepeatModeler (Flynn et al., 2020) to provide a more comprehensive repeat library for masking SatDNA in assembled genomes.

## 4 METHODS

### 4.1 Running SRF for human assemblies

We counted 171-mers occurring 20 times or more with KMC, using command line kmc –fm –k171 –ci20 –cs100000 and extracted the 171-mer counts with kmc_dump. SRF is directly applied to the output of kmc_dump output in the default setting.

### 4.2 Running SRF on sequence reads

We estimated the approximate read depth by dividing the total number of read bases by the number of bases in the reference genome or the corresponding read assembly. We counted 151-mers with kmc –fq –k151 –ciXX -cs1000000, where XX is 10 times the average read depth of each sample.

### 4.3 Estimating the abundance of SatDNA

We aligned reconstructed repeat units to HiFi reads or contigs with minimap2 (Li, 2018), using command line minimap2 –c –N1000000 –f1000 –r100,100 <(srfutils.js enlong srf.fa), where srfutils.js is a companion script along with the SRF tool. Option –N1000000 asks minimap2 to report up to a million hits per query sequence; –f1000 considers high-occurrence seeds; –r100,100 enables a small bandwidth of 100 bp during alignment.

After the alignment, we used srfutils.js paf2bed to filter poor alignments and to merge adjacent alignments, and then used srfutils.js bed2abun to calculate the abundance of each repeat unit.

### 4.4 Running TAREAN

For human CHM13, we used real reads short reads reads. We ran TAREAN with singularity exec ––bind ${PWD}:/data/shub://repeatexplorer/repex_tarean seqclust –p –c 32 –r 50000000. For *Drosorphila*, we simulated 125 bp paired-end reads from HiFi reads with dwgsim –N 146000 –1 125 –2 125 –y0 –e0 –E0 –r0 –F0 –R0. This command line did not add additional sequencing errors; the simulated reads only carried real sequencing errors on the original HiFi reads.

## 5 DATA ACCESS

The SRF implementation and associated analysis scripts are provided at https://github.com/lh3/srf. A modified TRF with an alternative command-line interface is available at https://github.com/lh3/TRF-mod. Assembled repeat units and their abundance estimates can be found at https://zenodo.org/record/7814465.

## Figures and Tables

**Table 1.** Features of user-facing tools for SatDNA reconstruction

Tool	Reads	Contigs	De novo	HORs

Alpha-CENTAURI	Yes	No	No	Yes
HiCAT	No	Yes	No	Yes
HORmon	No	Yes	No	Yes
NTRprism	No	Yes	Yes	Yes
SRF (this work)	Yes	Yes	Yes	Yes
TAREAN	Yes	No	Yes	No

“Reads”: whether the tool works with unassembled reads. “Contigs”: whether the tool works with high-quality contigs. “De novo”: whether the tool works without known monomer sequence as input. “HORs”: whether the tool can identify high-order repeats (HORs). References to these tools can be found in the Introduction section.

**Table 2.** Human chromosome-specific high-order alpha repeats (*α*HORs)

chr	HORmon ^a^ centromere	HiCAT ^b^ centromere	SRF (*k*=171) chromosome ^c^	SRF/171 assembly ^d^	SRF/171 HiFi reads ^d^	SRF/101 Illumina ^d^	SRF (*k*=171) HPRC assembly ^e^

1	6	2	6 (4.2); 11 (0.5)	6 (2.0)	6 (3.5)	2 (2.2)	6 [89]
2	4	4	4 (2.3)	4 (2.3)	4 (2.2)	4 (2.2)	4 [94]
3	17	17	17 (1.4)	17 (1.4)	17 (1.4)	17 (1.4)	17 [94]
4	19	19	19 (3.5)	19 (3.5)	19 (2.9)	19 (3.4)	19 [94]
5	6	12	8 (2.5)	8 (1.8)	4 (1.9)	missing	8 [43]; 4 [37]
6	18	18	18 (2.0)	18 (2.0)	18 (2.0)	18 (2.0)	18 [93]
7	6	6	6 (3.3)	6 (3.2)	6 (3.2)	6 (3.2)	6 [92]; 12 [2]
8	11	15	7 (1.1)	7 (1.1)	7 (1.0)	11 (1.0)	7 [61]; 8 [33]
9	7	11	4 (1.8)	4 (1.4)	11 (2.0)	4 (1.7)	4 [77]; 11 [17]
10	8	6	8 (2.1)	8 (2.1)	8 (1.7)	8 (2.1)	6 [66]; 8 [28]
11	5	5	5 (3.4)	5 (3.3)	5 (3.4)	5 (3.4)	5 [94]
12	8	8	8 (2.6)	8 (2.6)	8 (2.6)	8 (2.6)	8 [94]
13	11	7	4 (0.4)	4 (0.4)	7 (1.5)	7 (1.5)	4 [55]; 11 [23]; 7 [16]
14	8	8	8 (2.6)	missing	missing	missing	missing
15	11	15	11 (0.8); 20 (0.5)	11 (0.8)	11 (0.8)	11 (0.8)	11 [94]
16	10	10	10 (2.0)	10 (1.9)	10 (1.9)	missing	10 [94]
17	16	14	16 (3.3)	16 (3.3)	16 (3.5)	16 (3.5)	16 [56]; 13 [38]
18	12	12	8 (3.6)	8 (3.8)	12 (4.9)	missing	12 [66]; 8 [19]
19	2	2	4 (0.4); 2 (0.4)	missing	13 (0.5)	missing	13 [29]; 32 [4]
20	16	16	16 (2.1)	16 (2.1)	16 (2.1)	8 (0.5)	16 [94]
21	11	11	11 (0.3)	missing	missing	missing	missing
22	8	8	8 (2.9); 20 (0.5)	8 (2.8)	8 (2.6)	8 (2.9)	8 [94]
X	12	12	12 (3.1)	12 (3.1)	12 (3.1)	12 (3.1)	12 [76]
Y	34	No Y	34 (0.3)	34 (0.3)	No Y	No Y	34 [18]

a *α*HOR lengths in the monomer unit in the CHM13 v2.0 genome, retrieved from Kunyavskaya et al. (2022).

b length of “top 1” *α*HOR from each chromosome retrieved from Gao et al. (2022). Both HORmon and HiCAT were applicable to extracted centromeric sequences only.

c SRF applied to each CHM13 chromosome separately. In a format “*m* (*L*)”, *m* denotes the length of an HOR in the monomer unit and *L* is its span on the CHM13 assembly in megabases.

d SRF applied to CHM13 assembly, PacBio High-Fidelity (HiFi) reads and Illumina short reads, respectively. *k*=101 used for Illumina reads. CHM13 reads do not contain chrY.

e SRF applied to 94 phased haploid assemblies produced by the Human Pangenome Reference Consortium (HPRC). In a format “*m* [*n*]”, *m* is the monomer length and *n* is the number of samples with the HOR according to manual inspection.

**Table 3.** *A. thaliana* PacBio HiFi datasets

Sample	Strain	SRA Accession	Source

Col-0N	Col-0	ERR6210723	Naish et al. (2021)
Col-0R	Col-0	ERR8666127	Rabanal et al. (2022)
Ey15–2R	Eyach15–2	ERR8666125	Rabanal et al. (2022)

**Table 4.** HiFi datasets for non-model organisms

Species	Common name	Source

*A. bisporus*	cultivated mushroom	PRJEB52214
*A. ruthenus*	sterlet	PRJEB19273
*C. elaphus*	red deer	Pemberton et al. (2021)
*C. lupus*	grey wolf	Sinding et al. (2021)
*D. lineata*	orange-striped anemone	Wood et al. (2022)
*F. × ananassa*	royal royce strawberry	Hon et al. (2020)
*H. helix*	ivy	PRJEB47300
*L. sulphureus*	chicken mushroom	Wright et al. (2022)
*M. domestica*	apple	Könyves et al. (2022)
*M. meles*	Eurasian badger	Newman et al. (2022)
*M. sylvestris*	crab apple	Ruhsam et al. (2022)
*M. glacialis*	spiny starfish	Lawniczak et al. (2021)
*O. orca*	killer whale	Foote et al. (2022)
*O. sativa*	rice	SRR10238608
*P. pellucida*	blue-rayed limpet	Lawniczak et al. (2022)
*R. muscosa*	yellow-legged frog	Hon et al. (2020)
*V. atalanta*	red admiral butterfly	Lohse et al. (2021)
